# Exploring local and global stability of COVID-19 through numerical schemes

**DOI:** 10.1038/s41598-024-56938-x

**Published:** 2024-04-04

**Authors:** Nan Xiao, Hongyan Xu, Amjid Hussain Morani, Ali Shokri, Herbert Mukalazi

**Affiliations:** 1 School of Information and Engineering, Jingdezhen Ceramic University, Jingdezhen, 333403 China; 2 Department of Mathematics, Institute of Numerical Sciences, Gomal University, Dera Ismail Khan, 29050 KPK Pakistan; 3 Department of Mathematics, Faculty of Science, University of Maragheh, Maragheh, 83111-55181 Iran; 4 Department of Mathematics and Statistics, Kyambogo University, Kampala, Uganda

**Keywords:** Respiratory, Duration, Demonstrates, Outperforms, Confirmed, Simulations, Supported: unique, Computational biology and bioinformatics, Mathematics and computing

## Abstract

Respiratory sensitivity and pneumonia are possible outcomes of the coronavirus (COVID-19). Surface characteristics like temperature and sunshine affect how long the virus survives. This research article analyzes COVID-19 mathematical model behavior based on symptomatic and non-symptomatic individuals. In the reproductive model, the best result indicates the intensity of the epidemic. Our model remained stable at a certain point under controlled conditions after we evaluated a specific element. This approach is in place of traditional approaches such as Euler’s and Runge–Kutta’s. An unusual numerical approach known as the non-standard finite difference (NSFD) scheme is used in this article. This numerical approach gives us positivity. A dependable numerical analysis allowed us to evaluate different approaches and verify our theoretical results. Unlike the widely used Euler and RK4 approaches, we investigated the benefits of implementing NSFD schemes. By numerically simulating COVID-19 in a variety of scenarios, we demonstrated how our theoretical concepts work. The simulation findings support the usefulness of both approaches.

## Introduction

As humans, we are born with some active viruses and dormant microorganisms on Earth. Infectious diseases can be caused by viruses, bacteria, fungus, and arthropods. Currently, we are facing pandemic diseases such as HIV, COVID-19, Malaria, Influenza, Tuberculosis, Zika virus infection, Smallpox, measles, yellow fever, Cholera, and Leprosy all around the world^1–4^. Through history, humanity has faced numerous obstacles and problems, including world wars, infectious diseases, earthquakes, catastrophic floods, and climate change The effects of these incidents are gravely detrimental to the lives of individuals and civilizations alike. In addition to these challenges, infectious diseases continue to pose a significant threat to humanity, negatively affecting several countries' economies, education systems, and tourism industries. Among the deadly diseases that have affected humans are HIV, plague, and most recently, COVID-19^5–8^.

The first case of COVID-19 was detected on 19-12-2019 in Wuhan, China—a highly risky situation^9^. The COVID-19 infection rapidly spread worldwide, being more infectious and pandemic-prone than SARS^10–12^. Due to the rapid increase in cases, the World Health Organization declared COVID-19 a global pandemic on March 11th. Common symptoms of COVID-19 include fever, cough, congestion, fatigue, headaches, vomiting, diarrhea, shortness of breath, and a decrease in lymphocyte count^13,14^. To minimize human loss, crowds were restricted, and city lockdowns were implemented. In this situation, researchers and policymakers are working on finding a cure or vaccine for the aforementioned disease to stabilize and control it in the coming days. Some countries have successfully developed vaccines, and they are currently available^15–18^. The incubation period for COVID-19 typically spans five days, but it can extend up to fourteen days.

It has been noted that individuals differ in their susceptibility and ability to tolerate COVID-19. The fact that elderly individuals typically have worse clinical results than younger ones is also widely acknowledged^19–23^ According to earlier studies, COVID-19 patients who are older are more likely to pass away. The Susceptible-Infected-Recovered (SIR) epidemic model, developed by Chikina et al., simulates the effects of age-targeted mitigation activities for a COVID-19-like outbreak in the US by accounting for established age-contact patterns. Mortality and intensive care unit utilization can be considerably decreased by applying stringent age-targeted mitigation strategies^24–28^. An additional investigation verified that distinct dynamic patterns are displayed by COVID-19 epidemic processes across age and gender categories. In physics, chemistry, electromagnetics, and mechanics, fractional-order differential equations are commonly used. Fractional differential equations (FDEs) are widely used to identify various physical systems, control theory, simulate viscoelastic materials, and model numerous complex processes.

The propagation and dynamic behavior of COVID-19 have been analyzed using various mathematical models. These models can aid in comprehending the transmission of infectious diseases and developing preventative measures^29–32^. Peter et al.^33^ used real-world data to evaluate how various management strategies affect the transmission of COVID-19 among humans. The authors in^34–37^ investigated a mathematical model of COVID-19 that incorporates both the resistant compartment and a quarantine category. This model is different from earlier models discussed in the literature because of the resistive and quarantine classes. By improving the sources of control activities using mathematical models, we can successfully curb the spread of the disease. Furthermore, mathematical models have been employed by researchers to extensively investigate the novel coronavirus from different perspectives^38–42^.

Researchers focused on studying stability, numerical techniques, and local/global dynamics. In a related study, Lu, H^43^, and colleagues examined the impact of different COVID-19 management strategies using real-world data and a mathematical model. They used scaled conjugate gradient neural networks to analyze a nonlinear mathematical model for COVID-19. In this article the author's discussed the different scheme like Euler, RK-4 and a discrete NSFD scheme. The NSFD scheme was developed to study biological sustainability and other model features. The goal of using strategies like Euler, RK-4, and advanced NSFD was to control COVID-19 spread and assess threats to public health but the NSFD scheme gave us a positive result for all step size. We also discussed the local and global stability of endemic and disease-free equilibria for NSFD Scheme which were examined and showed to be suitable and unconditionally stable for the continuous model, providing precise and effective results.

The paper is structured in the following way: Sect. 1 introduces the mathematical model and comprehensively investigates its parameters. In Sect. 2, the model equilibria and the primary fundamental reproduction number are explained. Section 3, we discussed the comparative analysis of numerical scheme, in the subsections of this we create the Euler, Rk-4 and discrete NSFD scheme, and in the Subsections explores certain fundamental features, such as positivity and boundedness. Our study demonstrates that the NSFD scheme is a potent and efficient technique that accurately depicts the continuous model. We evaluate the local stability of both equilibria by employing the Schur-Cohn criterion in Subsection 3.4. In Subsection 3.5, we utilize the Lyapunov function theory to cover the global stability. Our theoretical findings are strengthened by numerical simulations. A conclusion section is included at the end of the manuscript that summarizes everything.

## Parameter explanation of the model

The system which we discussed^43^ consists of four differential equations. These equations represent the population $N\left(t\right)$, which is divided into four categories: susceptible $S(t)$, Infected $I(t)$, quarantined $Q(t)$, recovered $R(t)$ where $N\left(t\right)$= $S\left(t\right)+I\left(t\right)+Q\left(t\right)+R(t)$.

**Figure 1:**
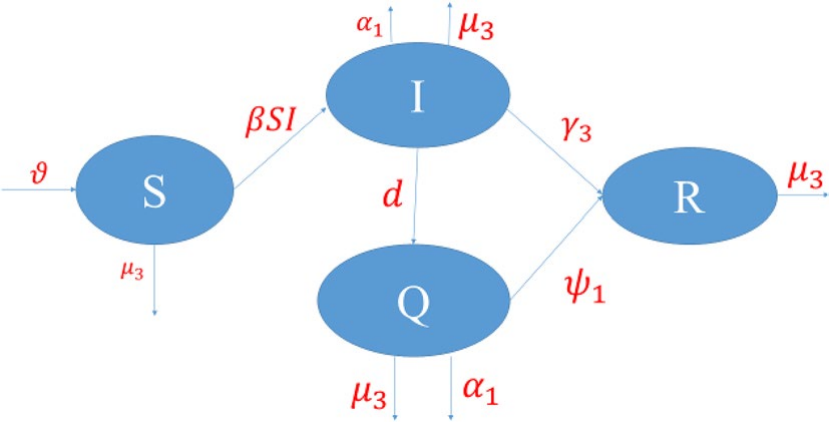
The flowchart of our model.

From the Fig. 1, we can derive a mathematical model for COVID-19.

$$\frac{dS}{dt}=\vartheta -\frac{\beta SI}{N}-{\mu }_{3}S$$

$$\frac{dI}{dt}=\frac{\beta SI}{N}-\left({\mu }_{3}+{\gamma }_{3}+{\alpha }_{1}+d\right)I$$

1 $$\frac{dQ}{dt}=dI-\left({\mu }_{3}+{\psi }_{1}+{\alpha }_{1}\right)Q$$

$$\frac{dR}{dt}={\gamma }_{3}I+{\psi }_{1}Q-{\mu }_{3}R$$

where $N$ is total population, so we reduced the $N$ from the above equation for simplified the model. Hence our system now becomes,

$$\frac{dS}{dt}=\vartheta -\beta SI-{\mu }_{3}S$$

$$\frac{dI}{dt}=\beta SI-\left({\mu }_{3}+{\gamma }_{3}+{\alpha }_{1}+d\right)I$$

2 $$\frac{dQ}{dt}=dI-\left({\mu }_{3}+{\psi }_{1}+{\alpha }_{1}\right)Q$$

$$\frac{dR}{dt}={\gamma }_{3}I+{\psi }_{1}Q-{\mu }_{3}R$$

### Parameters and their explanations

The following are some important concepts related to COVID-19:

**Table:** 

$\vartheta$	Natural population growth: This refers to the increase in population due to births and immigration, minus the decrease in population caused by deaths and emigration
$\beta$	Transition rate from I to R: This refers to the rate at which infected individuals recover from COVID-19 and become immune
$d$	Cure rate for quarantined individuals: This is the rate at which individuals who are quarantined due to COVID-19 recover and become immune
${\gamma }_{3}$	COVID-19 infection-related death rate: This is the rate of deaths caused by COVID-19 among infected individuals
${\alpha }_{1}$	Rate of COVID-19 death among those under quarantine: This is the rate of deaths caused by COVID-19 among individuals who are under quarantine
${\mu }_{3}$	Natural population decline: This refers to the decrease in population due to deaths and emigration, minus the increase in population caused by births and immigration
${\psi }_{1}$	Interval between infection and healing: This is the time it takes for an infected individual to recover from COVID-19 and become immune

## The equilibrium and the basic reproduction number (${{\varvec{R}}}_{0})$

### Modelling the equilibrium state

In order to find the disease-free equilibrium point (DFE), we can equate the model (2) to zero. It becomes easy to calculate DFE for model (2) when the DFE is represented by ${E}_{0}=\left(\frac{\vartheta }{{\mu }_{3}},\text{0,0},0\right)$. The given model (2) is instantaneously cracked for the state-run variables $S$,$I,Q,R,$ to invention the disease endemic equilibrium (DEE) point. If the DEE point is mentioned as ${E}^{*}\left({S}^{*},{I}^{*},{Q}^{*},{R}^{*}\right)$, then model (2) yields ${S}^{*}=\frac{\left(\vartheta -\beta {S}^{*}{I}^{*}\right)}{{\mu }_{3}}$, ${I}^{*}=\frac{\beta {S}^{*}{I}^{*}}{\left({\mu }_{3}+{\gamma }_{3}+{\alpha }_{1}+d\right)}$ , ${Q}^{*}=\frac{d{I}^{*}}{\left({\mu }_{3}+{\psi }_{1}+{\alpha }_{1}\right)}$ , and ${R}^{*}=\frac{{\gamma }_{3}{I}^{*}+{\psi }_{1}{Q}^{*}}{{\mu }_{3}}$

### Basic reproduction number ${({\varvec{R}}}_{0})$

It's difficult to determine the exact number of secondary infections, as it depends on factors like the spread of the disease, population size, and duration of illness. However, epidemiological studies can provide a rough estimate called the basic reproduction number^44^. To calculate ${R}_{0}$, we operate Model (2), which includes the translation matrix V(x) and the transmission matrix F(x).

$$F\left(x\right)=\left[\begin{array}{c}\beta SI\\ 0\end{array}\right],\text{ and }V(x)=\left[\begin{array}{c}\left({\mu }_{3}+{\gamma }_{3}+{\alpha }_{1}+d\right)I\\ dI-\left({\mu }_{3}+{\psi }_{1}+{\alpha }_{1}\right)Q\end{array}\right].$$

As ${R}_{0}=\rho (F{V}^{-1})$, therefore simple calculation employs.

$${R}_{0}=\frac{\beta \vartheta }{{\mu }_{3}\left({\mu }_{3}+{\psi }_{1}+{\alpha }_{1}\right)}.$$

## Comparative analysis of schemes

In this article, our goal is to illustrate the superior performance of the NSFD scheme compared to other numerical schemes such as Euler Rk4. It has been noted that the Euler and Rk4 schemes exhibit divergence as the time step size increases. Conversely, the NSFD scheme maintains non-divergence even with considerably larger time step sizes. Consequently, we can assert that the NSFD scheme stands out as the optimal choice among numerical schemes. In the ensuing discussion, we will thoroughly examine all three schemes Euler, Rk4, and NSFD and provide both numerical data and graphical representations to showcase their respective performances.

### The Euler scheme

The Euler scheme can be developed for the system (2) as shown below

$$\frac{{S}_{m+1}-{S}_{m}}{h}=\vartheta -\beta {S}_{m}{I}_{m}-{\mu }_{3}{S}_{m}$$

$${S}_{m,+1}-{S}_{m}=h\left(\vartheta -\beta {S}_{m}{I}_{m}-{\mu }_{3}{S}_{m}\right)$$

$${S}_{m+1}={S}_{m}+h\left(\vartheta -\beta {S}_{m}{I}_{m}-{\mu }_{3}{S}_{m}\right).$$

Similarly,

$$\frac{{I}_{m+1}-{I}_{m}}{h}=\left(\beta {S}_{m}{I}_{m}-\left({\mu }_{3}+{\gamma }_{3}+{\alpha }_{1}+d\right){I}_{m}\right)$$

$${I}_{m+1}-{I}_{m}=h\left(\beta {S}_{m}{I}_{m}-\left({\mu }_{3}+{\gamma }_{3}+{\alpha }_{1}+d\right){I}_{m}\right)$$

$${I}_{m+1}={I}_{m}+h\left(\beta {S}_{m}{I}_{m}-\left({\mu }_{3}+{\gamma }_{3}+{\alpha }_{1}+d\right){I}_{m}\right),$$

and

$$\frac{{Q}_{m+1}-{Q}_{m}}{h}=(d{I}_{m}-\left({\mu }_{3}+{\psi }_{1}+{\alpha }_{1}\right){Q}_{m})$$

$${Q}_{m+1}-{q}_{m}=h(d{I}_{m}-\left({\mu }_{3}+{\psi }_{1}+{\alpha }_{1}\right){Q}_{m})$$

$${Q}_{m+1}={Q}_{m}+h(d{I}_{m}-\left({\mu }_{3}+{\psi }_{1}+{\alpha }_{1}\right){Q}_{m}),$$

and

$$\frac{{R}_{m+1}-{R}_{m}}{h}=\left({\gamma }_{3}{I}_{m}+{\psi }_{1}{Q}_{m}-{\mu }_{3}{R}_{m}\right)$$

$${R}_{m+1}-{R}_{m}==h\left({\gamma }_{3}{I}_{m}+{\psi }_{1}{Q}_{m}-{\mu }_{3}{R}_{m}\right)$$

$${R}_{m+1}={R}_{m}+h\left({\gamma }_{3}{I}_{m}+{\psi }_{1}{Q}_{m}-{\mu }_{3}{R}_{m}\right).$$

### The RK-4 scheme

Our challenges often use the RK-4 scheme unless otherwise noted. The RK-4 scheme was developed for system (2).we take $S={A}_{1}, I={B}_{1}, Q={C}_{1} {\text{and}} R={D}_{1}$, then.

**Itération 1**

$${A}_{1}={\text{h}}\left(\left(\vartheta -\beta {S}_{m}{I}_{m}-{\mu }_{3}{S}_{m}\right)\right)$$

$${B}_{1}=h\left(\beta {S}_{m}{I}_{m}-\left({\mu }_{3}+{\gamma }_{3}+{\alpha }_{1}+d\right){I}_{m}\right)$$

$${C}_{1}=h(d{I}_{m}-\left({\mu }_{3}+{\psi }_{1}+{\alpha }_{1}\right){Q}_{m})$$

$${D}_{1}=h\left({\gamma }_{3}{I}_{m}+{\psi }_{1}{Q}_{m}-{\mu }_{3}{R}_{m}\right).$$

**Itération 2**

$${A}_{2}={\text{h}}\left(\left(\vartheta -\beta {(S}_{m}+\frac{{A}_{1}}{2})({I}_{m}+\frac{{B}_{1}}{2}) -{\mu }_{3}({S}_{m}+\frac{{A}_{1}}{2}\right)\right)$$

$${B}_{2}=h\left(\beta {(S}_{m}+\frac{{A}_{1}}{2}\right)\left({I}_{m}+\frac{{B}_{1}}{2}\right)-\left({\mu }_{3}+{\gamma }_{3}+{\alpha }_{1}+d\right)\left({I}_{m}+\frac{{B}_{1}}{2}\right)$$

$${C}_{2}=\left(d{(I}_{m}+\frac{{B}_{1}}{2}\right)-\left({\mu }_{3}+{\psi }_{1}+{\alpha }_{1}\right)\left({Q}_{m}+\frac{{C}_{2}}{2}\right)$$

$${D}_{2}=\left({\gamma }_{3}({I}_{m}+\frac{{B}_{1}}{2}) +{\psi }_{1}({Q}_{m}+\frac{{C}_{2}}{2})-{\mu }_{3}\left({R}_{m}+\frac{{D}_{4}}{2}\right)\right)$$

**Itération 3**

$${A}_{3}=h\left(\left(\vartheta -\beta {(S}_{m}+\frac{{A}_{2}}{2})({I}_{m}+\frac{{B}_{2}}{2}) -{\mu }_{3}({S}_{m}+\frac{{A}_{2}}{2}\right)\right)$$

$${B}_{3}=h\left(\beta {(S}_{m}+\frac{{A}_{2}}{2}\right)\left({I}_{m}+\frac{{B}_{2}}{2}\right)-\left({\mu }_{3}+{\gamma }_{3}+{\alpha }_{1}+d\right)\left({I}_{m}+\frac{{B}_{1}}{2}\right)$$

$${C}_{3}=\left(d{(I}_{m}+\frac{{B}_{1}}{2}\right)-\left({\mu }_{3}+{\psi }_{1}+{\alpha }_{1}\right)\left({Q}_{m}+\frac{{C}_{2}}{2}\right)$$

$${D}_{3}=\left({\gamma }_{3}({I}_{m}+\frac{{B}_{1}}{2}) +{\psi }_{1}({Q}_{m}+\frac{{C}_{2}}{2})-{\mu }_{3}\left({R}_{m}+\frac{{D}_{3}}{2}\right)\right).$$

**Itération 4**

$${A}_{4}=h\left(\left(\vartheta -\beta {(S}_{m}+{A}_{3})({I}_{m}+{B}_{3})-{\mu }_{3}({S}_{m}+{A}_{3})\right)\right)$$

$${B}_{4}=h\left(\beta {(S}_{m}+{A}_{3})\left({I}_{m}+{B}_{3}\right)-\left({\mu }_{3}+{\gamma }_{3}+{\alpha }_{1}+d\right)({I}_{m}+{B}_{3}\right)$$

$${C}_{4}=h(d{(I}_{m}+{B}_{3})-\left({\mu }_{3}+{\psi }_{1}+{\alpha }_{1}\right)({Q}_{m}+{C}_{3})).$$

$${D}_{3}=h\left({\gamma }_{3}({I}_{m}+{B}_{3})+{\psi }_{1}({Q}_{m}+{C}_{3})-{\mu }_{3}\left({R}_{m}+{D}_{3}\right)\right).$$

The final stage is

$$\Delta {z}_{1}=\frac{1}{6}({A}_{1}+2{A}_{2}{+2A}_{3}{+A}_{4})$$

$$\Delta {z}_{2}=\frac{1}{6}({B}_{1}+2{B}_{2}+2{B}_{3}+{B}_{4})$$

$$\Delta {z}_{3}=\frac{1}{6}({C}_{1}+2{C}_{2}+2{C}_{3}+{C}_{4})$$

$$\Delta {z}_{4}=\frac{1}{6}({D}_{1}+2{D}_{2}+2{D}_{3}+{D}_{4}),$$

Where $\Delta {z}_{1},\Delta {z}_{2},\Delta {z}_{3}{\text{and}} \Delta {z}_{4}$ are the weighted of ${A}_{1},{A}_{2}{,A}_{3}{,A}_{4}{,B}_{1},{B}_{2 },{B}_{3},{B}_{4},{C}_{1},{C}_{2}, {C}_{3},{C}_{4}{,D}_{1}$, ${D}_{2},{D}_{3 }\text{and }{D}_{4}$.

The general form is.

$${z}_{m+1}={z}_{m}+\Delta z.$$

Finally, we get

$${S}_{m+1}={S}_{m}+\frac{1}{6}({A}_{1}+2{A}_{2}{+2A}_{3}{+A}_{4})$$

$${I}_{m+1}={I}_{m}+\frac{1}{6}({B}_{1}+2{B}_{2}+2{B}_{3}+{B}_{4})$$

$${Q}_{m+1}={Q}_{m}+\frac{1}{6}({C}_{1}+2{C}_{2}+2{C}_{3}+{C}_{4})$$

$${R}_{m+1}={R}_{m}+\frac{1}{6}({D}_{1}+2{D}_{2}+2{D}_{3}+{D}_{4}).$$

### The NSFD scheme

The value of h (time step size) represents the numerical approximations of $S\left(t\right)$,$I\left(t\right)$,$Q(t)$, ${\text{and}} R\left(t\right)$ at $t=mh$ as per model (2). To denote these numerical estimates, we use the notation ${S}_{n}, {I}_{n},{Q}_{n}, {R}_{n}$, where $n$ is a non-negative integer^45,46^. Subsequently, model (2) enables us to express.

$$\frac{{S}_{m+1}-{S}_{m}}{h}=\vartheta -\beta {S}_{m+1}{I}_{m}-{\mu }_{3}{S}_{m+1}$$

$$\frac{{I}_{m+1}-{I}_{m}}{h}=\beta {{S}_{m}}_{+1}{I}_{m}-\left({\mu }_{3}+{\gamma }_{3}+{\alpha }_{1}+d\right){I}_{m+1}$$

3 $$\frac{{Q}_{m+1}-{Q}_{m}}{h}=d{I}_{m+1}-\left({\mu }_{3}+{\psi }_{1}+{\alpha }_{1}\right){Q}_{m+1}$$

$$\frac{{R}_{m+1}-{R}_{m}}{h}={\gamma }_{3}{I}_{m+1}+{\psi }_{1}{Q}_{m+1}-{\mu }_{3}{R}_{m+1}.$$

The explicit form of the NSFD scheme (3) becomes after simplification.

$${S}_{m+1}=\frac{{S}_{m}+h\vartheta }{1+h\left(\beta {I}_{m}+{\mu }_{3}\right)}$$

$${I}_{m+1}=\frac{\left({I}_{m}+h\beta {{S}_{m}}_{+1}{I}_{m}\right)}{1+h\left({\mu }_{3}+{\gamma }_{3}+{\alpha }_{1}+d\right)}$$

4 $${Q}_{m+1}=\frac{\left({Q}_{m}+hd{I}_{m+1}\right)}{\left(1+h\left({\mu }_{3}+{\psi }_{1}+{\alpha }_{1}\right)\right)}$$

$${R}_{m+1} =\frac{{R}_{m}+h{\gamma }_{3}{I}_{m+1}+h{\psi }_{1}{Q}_{m+1}}{\left(1+h{\mu }_{3}\right)}$$

### Positivity and boundedness of NSFD scheme

We suppose that ${S}_{0}\ge 0,{I}_{0}\ge 0,{Q}_{0}\ge 0,{R}_{0}\ge 0,$., which are the starting values that constitute the discrete scheme (4), are all non-negative. According to the underlying assumptions, these variables also have nonnegative estimated amounts ${S}_{m}\ge 0{, I}_{m}\ge 0,{Q}_{m}\ge 0,{R}_{m}\ge 0$ Hence, explanations of NSFD scheme (4) suggest the positivity of scheme (4), i.e. ${S}_{m+1}\ge 0,{I}_{m+1}\ge 0,{Q}_{m+1}\ge 0,{R}_{m+1}\ge 0$. In deliberate the boundedness of results of the NSFD arrangement (4), we consider ${W}_{n}={S}_{m}+{I}_{m}+{Q}_{m}+{R}_{m}$. Then.

$$\frac{{W}_{m+1}-{W}_{m}}{h}=(\vartheta -{\mu }_{3}){W}_{m+1},$$

I.e.

$$(1+{\mu }_{3}){W}_{m+1}=h\vartheta +{W}_{n}.$$

Therefore, we get

$${W}_{m+1}\le \frac{h\vartheta }{(1+{\mu }_{3})}+\frac{{W}_{n}}{(1+{\mu }_{3})}\Leftrightarrow h\vartheta {\sum }_{n+1}^{p}{\left(\frac{1}{(1+{\mu }_{3})}\right)}^{n}+{W}_{0}{\left(\frac{1}{(1+{\mu }_{3})}\right)}^{p}.$$

If $0<W\left(0\right)<\frac{\vartheta }{{\mu }_{3}}$, then by Gromwell’s inequality is used to produce.

$${W}_{n}\le \frac{\vartheta }{{\mu }_{3}}(1-\frac{1}{{\left(1+h{\mu }_{3}\right)}^{p}})+{W}_{0}{\left(\frac{1}{(1+h{\mu }_{3}}\right)}^{p}=\frac{\vartheta }{{\mu }_{3}}+\left({W}_{0}-\frac{\vartheta }{{\mu }_{3}}\right){\left(\frac{1}{(1+h{\mu }_{3}}\right)}^{p}.$$

Since ${\left(\frac{1}{(1+h{\mu }_{3}}\right)}^{p}<1$, so we gain $W_{n} \to \frac{\vartheta }{{\mu_{3} }}$ as $m \to \infty$. This indicates that the feasible region becomes and the solutions of system (4) are bounded.

$$F=\left\{\left({S}_{m}+{I}_{m}+{Q}_{m}+{R}_{m}\right):0\le {S}_{n}+{I}_{n}+{Q}_{n}+{R}_{n}\le \frac{\vartheta }{{\mu }_{3}}\right\}.$$

We evaluate the local stability of both equilibria in the NSFD scheme (4).

$${S}_{m+1}=\frac{{S}_{m}+h\vartheta }{1+h\left(\beta {I}_{m}+{\mu }_{3}\right)}={f}_{1}$$

$${I}_{m+1}=\frac{\left({I}_{m}+h\beta {{S}_{m}}_{+1}{I}_{m}\right)}{1+h\left({\mu }_{3}+{\gamma }_{3}+{\alpha }_{1}+d\right)}={f}_{2}$$

5 $${Q}_{m+1}=\frac{\left({Q}_{m}+hd{I}_{m+1}\right)}{\left(1+h\left({\mu }_{3}+{\psi }_{1}+{\alpha }_{1}\right)\right)}={f}_{3}$$

$${R}_{m+1} =\frac{{R}_{m}+h{\gamma }_{3}{I}_{m+1}+h{\psi }_{1}{Q}_{m+1}}{\left(1+h{\mu }_{3}\right)}={f}_{4}$$

### Local stability of equilibria

Lemma 1 follows. We will apply the Schur-Cohn criterion^47,48^ to demonstrate that the DFE point is LAS.

#### Lemma 1

The roots of ${\mathcal{L}}^{2}-B\mathcal{L}+C=0$ assurance $\left|{\mathcal{L}}_{p}\right|<1 \text{for } p=\text{1,2}$, $\Leftrightarrow$ the necessities assumed in the succeeding remain satisfied.

1. $$C<1$$,
2. $$1+B+C>0$$,
3. $$1-B+C>0$$,

anywhere $B$ represents trace and $C$ shows determinant of the Jacobian matrix.

#### Theorem 1

$\forall$ $h>0$, the DFE point is LAS for NSFD model (4) whenever ${R}_{0}<1$.

#### Proof On

The information, we can express the Jacobian matrix in the following manner:

6 $$J\left(S,I,Q,R\right)=\left[\begin{array}{cccc}\frac{\partial {f}_{1}}{\partial S}& \frac{\partial {f}_{1}}{\partial I}& \frac{\partial {f}_{1}}{\partial Q}& \frac{\partial {f}_{1}}{\partial R}\\ \frac{\partial {f}_{2}}{\partial S}& \frac{\partial {f}_{2}}{\partial I}& \frac{\partial {f}_{2}}{\partial Q}& \frac{\partial {f}_{2}}{\partial R}\\ \frac{\partial {f}_{3}}{\partial S}& \frac{\partial {f}_{3}}{\partial I}& \frac{\partial {f}_{3}}{\partial Q}& \frac{\partial {f}_{3}}{\partial R}\\ \frac{\partial {f}_{4}}{\partial S}& \frac{\partial {f}_{4}}{\partial I}& \frac{\partial {f}_{4}}{\partial Q}& \frac{\partial {f}_{4}}{\partial R}\end{array}\right],$$

In (5), ${f}_{1}$, ${f}_{2}$, ${f}_{3}$, and ${f}_{4}$ (4) are provided. As you can see, every derivative used in (6) is noted here.

$$\begin{aligned} & \frac{\partial {f}_{1}}{\partial S}=\frac{1}{1+h\left(\beta {I}_{m}+{\mu }_{3}\right)}, \frac{\partial {f}_{1}}{\partial I}=\frac{-h\vartheta }{{\left(1+h\left(\beta {I}_{m}+{\mu }_{3}\right)\right)}^{2}},\frac{\partial {f}_{1}}{\partial Q}=0,\frac{\partial {f}_{1}}{\partial R}=0,\frac{\partial {f}_{2}}{\partial S}=0, \\ & \frac{\partial {f}_{2}}{\partial I}=\frac{1}{1+h\left({\mu }_{3}+{\gamma }_{3}+{\alpha }_{1}+d\right)} ,\frac{\partial {f}_{2}}{\partial Q}=0,\frac{\partial {f}_{2}}{\partial R}=0,\frac{\partial {f}_{3}}{\partial S}=0,\frac{\partial {f}_{3}}{\partial I}=\frac{hd}{\left(1+h\left({\mu }_{3}+{\psi }_{1}+{\alpha }_{1}\right)\right)},\\ &\frac{\partial {f}_{3}}{\partial Q}=\frac{1}{1+h\left({\mu }_{3}+{\psi }_{1}+{\alpha }_{1}\right)},\frac{\partial {f}_{3}}{\partial R}=0,\frac{\partial {f}_{4}}{\partial S}=0, \frac{\partial {f}_{4}}{\partial I}=\frac{h{\gamma }_{3}}{\left(1+h{\mu }_{3}\right)},\frac{\partial {f}_{4}}{\partial Q}=\frac{h{\psi }_{1}}{\left(1+h{\mu }_{3}\right)},\frac{\partial {f}_{4}}{\partial R}=\frac{1}{\left(1+h{\mu }_{3}\right)}\end{aligned}$$

When all of the aforementioned derivatives are entered into Eq. (6), we get

7 $$J=\left[\begin{array}{cccc}\frac{1}{1+h\left(\beta {I}_{m}+{\mu }_{3}\right)}& \frac{-h\vartheta }{{\left(1+h\left(\beta {I}_{m}+{\mu }_{3}\right)\right)}^{2}}& 0& 0\\ 0& \frac{1}{1+h\left({\mu }_{3}+{\gamma }_{3}+{\alpha }_{1}+d\right)}& 0& 0\\ 0& \frac{hd}{\left(1+h\left({\mu }_{3}+{\psi }_{1}+{\alpha }_{1}\right)\right)}& \frac{1}{1+h\left({\mu }_{3}+{\psi }_{1}+{\alpha }_{1}\right)}& 0\\ 0& \frac{h{\gamma }_{3}}{\left(1+h{\mu }_{3}\right)}& \frac{h{\psi }_{1}}{\left(1+h{\mu }_{3}\right)}& \frac{1}{\left(1+h{\mu }_{3}\right)}\end{array}\right].$$

By putting the ${E}_{0}=\left(\frac{\vartheta }{{\mu }_{3}},\text{0,0},0\right)$, the matrix (7).

$$J\left({E}_{0}\right)=\left[\begin{array}{cccc}\frac{1}{1+h{\mu }_{3}}& \frac{-h\vartheta }{{\left(1+h{\mu }_{3}\right)}^{2}}& 0& 0\\ 0& \frac{1}{1+h\left({\mu }_{3}+{\gamma }_{3}+{\alpha }_{1}+d\right)}& 0& 0\\ 0& \frac{hd}{\left(1+h\left({\mu }_{3}+{\psi }_{1}+{\alpha }_{1}\right)\right)}& \frac{1}{1+h\left({\mu }_{3}+{\psi }_{1}+{\alpha }_{1}\right)}& 0\\ 0& \frac{h{\gamma }_{3}}{\left(1+h{\mu }_{3}\right)}& \frac{h{\psi }_{1}}{\left(1+h{\mu }_{3}\right)}& \frac{1}{\left(1+h{\mu }_{3}\right)}\end{array}\right].$$

To explain the eigenvalues, we rely on the following assumptions:

$$\left|J\left({E}_{0}\right)-\mathcal{L}I\right|=0,$$

i.e.

8 $$\left|\begin{array}{cccc}\frac{1}{1+h{\mu }_{3}}-\mathcal{L}& \frac{-h\vartheta }{{\left(1+h{\mu }_{3}\right)}^{2}}& 0& 0\\ 0& \frac{1}{1+h\left({\mu }_{3}+{\gamma }_{3}+{\alpha }_{1}+d\right)}-\mathcal{L}& 0& 0\\ 0& \frac{hd}{\left(1+h\left({\mu }_{3}+{\psi }_{1}+{\alpha }_{1}\right)\right)}& \frac{1}{1+h\left({\mu }_{3}+{\psi }_{1}+{\alpha }_{1}\right)}-\mathcal{L}& 0\\ 0& \frac{h{\gamma }_{3}}{\left(1+h{\mu }_{3}\right)}& \frac{h{\psi }_{1}}{\left(1+h{\mu }_{3}\right)}& \frac{1}{\left(1+h{\mu }_{3}\right)}-\mathcal{L}\end{array}\right|=0.$$

After the simplify, (8) yields

9 $$\left(\frac{1}{1+h{\mu }_{3}}-{\mathcal{L}}_{1}\right)\left(\frac{1}{\left(1+h{\mu }_{3}\right)}-{\mathcal{L}}_{2}\right)\left|\begin{array}{cc}\frac{1}{1+h\left({\mu }_{3}+{\gamma }_{3}+{\alpha }_{1}+d\right)}-\mathcal{L}& 0\\ \frac{hd}{\left(1+h\left({\mu }_{3}+{\psi }_{1}+{\alpha }_{1}\right)\right)}& \frac{1}{1+h\left({\mu }_{3}+{\psi }_{1}+{\alpha }_{1}\right)}-\mathcal{L}\end{array}\right|=0.$$

The Eq. (9) provides ${\mathcal{L}}_{1}=\frac{1}{1+h{\mu }_{3}}<1,{\mathcal{L}}_{2}=\frac{1}{\left(1+h{\mu }_{3}\right)}<1.$

The other eigenvalues, we take

$$\left|\begin{array}{cc}\frac{1}{1+h\left({\mu }_{3}+{\gamma }_{3}+{\alpha }_{1}+d\right)}-\mathcal{L}& 0\\ \frac{hd}{\left(1+h\left({\mu }_{3}+{\psi }_{1}+{\alpha }_{1}\right)\right)}& \frac{1}{1+h\left({\mu }_{3}+{\psi }_{1}+{\alpha }_{1}\right)}-\mathcal{L}\end{array}\right|=0,$$

i.e.

10 $${\mathcal{L}}^{2}-\mathcal{L}\left(\frac{1}{1+h\left({\mu }_{3}+{\gamma }_{3}+{\alpha }_{1}+d\right)}+\frac{1}{1+h\left({\mu }_{3}+{\psi }_{1}+{\alpha }_{1}\right)}\right)+\frac{1}{1+h\left({\mu }_{3}+{\gamma }_{3}+{\alpha }_{1}+d\right)}\frac{1}{1+h\left({\mu }_{3}+{\psi }_{1}+{\alpha }_{1}\right)}=0$$

Comparing Eq. (10) with ${\mathcal{L}}^{2}-B\mathcal{L}+C=0$, we get $B=\left(\frac{1}{1+h\left({\mu }_{3}+{\gamma }_{3}+{\alpha }_{1}+d\right)}+\frac{1}{1+h\left({\mu }_{3}+{\psi }_{1}+{\alpha }_{1}\right)}\right)$ and $C=\frac{1}{1+h\left({\mu }_{3}+{\gamma }_{3}+{\alpha }_{1}+d\right)}\frac{1}{1+h\left({\mu }_{3}+{\psi }_{1}+{\alpha }_{1}\right)}$.

If $${R}_{0}<1,$$

1. $$C=\frac{1}{1+h\left({\mu }_{3}+{\gamma }_{3}+{\alpha }_{1}+d\right)}\frac{1}{1+h\left({\mu }_{3}+{\psi }_{1}+{\alpha }_{1}\right)}<1$$.
2. $$1+B+C=1+\frac{1}{1+h\left({\mu }_{3}+{\gamma }_{3}+{\alpha }_{1}+d\right)}+\frac{1}{1+h\left({\mu }_{3}+{\psi }_{1}+{\alpha }_{1}\right)}+\frac{1}{1+h\left({\mu }_{3}+{\gamma }_{3}+{\alpha }_{1}+d\right)}\frac{1}{1+h\left({\mu }_{3}+{\psi }_{1}+{\alpha }_{1}\right)}>0$$.
3. $$1-B+C=1-\frac{1}{1+h\left({\mu }_{3}+{\gamma }_{3}+{\alpha }_{1}+d\right)}-\frac{1}{1+h\left({\mu }_{3}+{\psi }_{1}+{\alpha }_{1}\right)}+\frac{1}{1+h\left({\mu }_{3}+{\gamma }_{3}+{\alpha }_{1}+d\right)}\frac{1}{1+h\left({\mu }_{3}+{\psi }_{1}+{\alpha }_{1}\right)}>0$$.

The Schur-Cohn criterion is therefore satisfied whenever ${R}_{0}<1$. As a result, NSFD scheme (4) DFE point ${E}_{0}$ is LAS if ${R}_{0}<1$.

#### Theorem 2

$h>0$, the DEE point is LAS for NSFD model (4) whenever ${R}_{0}>1$.

#### Proof

We develop it as follows, following a similar procedure to that by which we acquired the Jacobian matrix in Theorem 1

11 $$J=\left[\begin{array}{cccc}\frac{1}{1+h\left(\beta {I}_{m}+{\mu }_{3}\right)}& \frac{-h\vartheta }{{\left(1+h\left(\beta {I}_{m}+{\mu }_{3}\right)\right)}^{2}}& 0& 0\\ 0& \frac{1}{1+h\left({\mu }_{3}+{\gamma }_{3}+{\alpha }_{1}+d\right)}& 0& 0\\ 0& \frac{hd}{\left(1+h\left({\mu }_{3}+{\psi }_{1}+{\alpha }_{1}\right)\right)}& \frac{1}{1+h\left({\mu }_{3}+{\psi }_{1}+{\alpha }_{1}\right)}& 0\\ 0& \frac{h{\gamma }_{3}}{\left(1+h{\mu }_{3}\right)}& \frac{h{\psi }_{1}}{\left(1+h{\mu }_{3}\right)}& \frac{1}{\left(1+h{\mu }_{3}\right)}\end{array}\right].$$

By placing DEE point ${E}^{*}$, Eq. (11)

$$J\left({E}^{*}\right)=\left[\begin{array}{cccc}\frac{1}{1+h\left(\beta {I}_{m}^{*}+{\mu }_{3}\right)}& \frac{-h\vartheta }{{\left(1+h\left(\beta {I}_{m}^{*}+{\mu }_{3}\right)\right)}^{2}}& 0& 0\\ 0& \frac{1}{1+h\left({\mu }_{3}+{\gamma }_{3}+{\alpha }_{1}+d\right)}& 0& 0\\ 0& \frac{hd}{\left(1+h\left({\mu }_{3}+{\psi }_{1}+{\alpha }_{1}\right)\right)}& \frac{1}{1+h\left({\mu }_{3}+{\psi }_{1}+{\alpha }_{1}\right)}& 0\\ 0& \frac{h{\gamma }_{3}}{\left(1+h{\mu }_{3}\right)}& \frac{h{\psi }_{1}}{\left(1+h{\mu }_{3}\right)}& \frac{1}{\left(1+h{\mu }_{3}\right)}\end{array}\right].$$

To discuss the eigenvalues, we take

$$\left|J\left({E}^{*}\right)-\Gamma I\right|=0,$$

i.e.

12 $$\left|\begin{array}{cccc}\frac{1}{1+h\left(\beta {I}_{m}^{*}+{\mu }_{3}\right)}-\mathcal{L}& \frac{-h\vartheta }{{\left(1+h\left(\beta {I}_{m}^{*}+{\mu }_{3}\right)\right)}^{2}}& 0& 0\\ 0& \frac{1}{1+h\left({\mu }_{3}+{\gamma }_{3}+{\alpha }_{1}+d\right)}-\mathcal{L}& 0& 0\\ 0& \frac{hd}{\left(1+h\left({\mu }_{3}+{\psi }_{1}+{\alpha }_{1}\right)\right)}& \frac{1}{1+h\left({\mu }_{3}+{\psi }_{1}+{\alpha }_{1}\right)}-\mathcal{L}& 0\\ 0& \frac{h{\gamma }_{3}}{\left(1+h{\mu }_{3}\right)}& \frac{h{\psi }_{1}}{\left(1+h{\mu }_{3}\right)}& \frac{1}{\left(1+h{\mu }_{3}\right)}-\mathcal{L}\end{array}\right|=0.$$

After simplification, (12) yields

13 $$\left(\frac{1}{1+h\left(\beta {I}_{m}^{*}+{\mu }_{3}\right)}-\mathcal{L}\right)\left(\frac{1}{\left(1+h{\mu }_{3}\right)}-\mathcal{L}\right)\left|\begin{array}{cc}\frac{1}{1+h\left({\mu }_{3}+{\gamma }_{3}+{\alpha }_{1}+d\right)}-\mathcal{L}& 0\\ \frac{hd}{\left(1+h\left({\mu }_{3}+{\psi }_{1}+{\alpha }_{1}\right)\right)}& \frac{1}{1+h\left({\mu }_{3}+{\psi }_{1}+{\alpha }_{1}\right)}-\mathcal{L}\end{array}\right|=0.$$

It has two roots (13) are ${\mathcal{L}}_{1}=\frac{1}{1+h\left(\beta {I}_{m}^{*}+{\mu }_{3}\right)}<1$, and ${\mathcal{L}}_{2}=\frac{1}{\left(1+h{\mu }_{3}\right)}<1$. To find other eigenvalues, we take

$$\left|\begin{array}{cc}\frac{1}{1+h\left({\mu }_{3}+{\gamma }_{3}+{\alpha }_{1}+d\right)}-\mathcal{L}& 0\\ \frac{hd}{\left(1+h\left({\mu }_{3}+{\psi }_{1}+{\alpha }_{1}\right)\right)}& \frac{1}{1+h\left({\mu }_{3}+{\psi }_{1}+{\alpha }_{1}\right)}-\mathcal{L}\end{array}\right|=0,$$

14 $$\begin{aligned} & {\text{i}}.\text{e }{\mathcal{L}}^{2}-\mathcal{L}\left(\frac{1}{1+h\left({\mu }_{3}+{\gamma }_{3}+{\alpha }_{1}+d\right)}+\frac{1}{1+h\left({\mu }_{3}+{\psi }_{1}+{\alpha }_{1}\right)}\right)\\ &\quad+\frac{1}{1+h\left({\mu }_{3}+{\gamma }_{3}+{\alpha }_{1}+d\right)}\frac{1}{1+h\left({\mu }_{3}+{\psi }_{1}+{\alpha }_{1}\right)}=0=0.\end{aligned}$$

Comparing (14) with (10), we get $B=\left(\frac{1}{1+h\left({\mu }_{3}+{\gamma }_{3}+{\alpha }_{1}+d\right)}+\frac{1}{1+h\left({\mu }_{3}+{\psi }_{1}+{\alpha }_{1}\right)}\right)$ and $=$ $\frac{1}{1+h\left({\mu }_{3}+{\gamma }_{3}+{\alpha }_{1}+d\right)}\frac{1}{1+h\left({\mu }_{3}+{\psi }_{1}+{\alpha }_{1}\right)}$. If ${R}_{0}>1,$ then.

1. $$C=\frac{1}{1+h\left({\mu }_{3}+{\gamma }_{3}+{\alpha }_{1}+d\right)}\frac{1}{1+h\left({\mu }_{3}+{\psi }_{1}+{\alpha }_{1}\right)}<1.$$
2. $$1+B+C=1+\frac{1}{1+h\left({\mu }_{3}+{\gamma }_{3}+{\alpha }_{1}+d\right)}+\frac{1}{1+h\left({\mu }_{3}+{\psi }_{1}+{\alpha }_{1}\right)}+\frac{1}{1+h\left({\mu }_{3}+{\gamma }_{3}+{\alpha }_{1}+d\right)}\frac{1}{1+h\left({\mu }_{3}+{\psi }_{1}+{\alpha }_{1}\right)}>0.$$
3. $$1-B+C=1-\frac{1}{1+h\left({\mu }_{3}+{\gamma }_{3}+{\alpha }_{1}+d\right)}-\frac{1}{1+h\left({\mu }_{3}+{\psi }_{1}+{\alpha }_{1}\right)}+\frac{1}{1+h\left({\mu }_{3}+{\gamma }_{3}+{\alpha }_{1}+d\right)}\frac{1}{1+h\left({\mu }_{3}+{\psi }_{1}+{\alpha }_{1}\right)}>0.$$

Accordingly, when ${R}_{0}>1$, all of the longings of Schur-Cohn criterion quantified in Lemma 1 are pleased. So, on condition that that ${R}_{0}>1$, the DEE point ${E}^{*}$ of the NSFD scheme (4) is LAS.

### Global stability of equilibria

The process by which we obtained the Jacobian matrix in Theorem 1 is analogous to the one we use to derive it.

#### Theorem 3

$h>0$, the DFE point is GAS for NSFD model (4) ${R}_{0}\le 1$.

#### Proof

Construct a Lyapunov function.

$${P}_{n}\left({S}_{n}{, I}_{n},{Q}_{n},{R}_{n},\right)={S}^{0}H\left(\frac{{S}_{n}}{{S}^{0}}\right)+{\Psi }_{1}{I}_{n}+{\Psi }_{2}{Q}_{n}+{\Psi }_{3}{R}_{n},$$

where ${\Psi }_{i}>0$ for all $i=\text{1,2},\text{3,4}$. Hence, ${P}_{n}>0$ for all ${S}_{n}>0, {I}_{n}>0, {Q}_{n}>0, {\text{and}} {R}_{n}$ In addition, ${P}_{n}=0,$ if and only if ${S}_{n}={S}^{0},{I}_{n}={I}^{0}, {Q}_{n}={Q}^{0}, {\text{and}}{R}_{n}={R}^{0}$.

We take

$$\Delta {P}_{n}={P}_{n+1}-{P}_{n},$$

i.e.

$$\Delta {P}_{n}={S}^{0}F\left(\frac{{S}_{m+1}}{{S}^{0}}\right)+{\Psi }_{1}{I}_{m+1}+{\Psi }_{2}{Q}_{m+1}+{\Psi }_{3}{R}_{m+1}-\left({S}^{0}F\left(\frac{{S}_{m}}{{S}^{0}}\right)+{\Psi }_{1}{I}_{m}+{\phi }_{3}{Q}_{m}+{\Psi }_{4}{R}_{m}\right).$$

15 $$={S}^{0}\left(\frac{{S}_{m+1}}{{S}^{0}}-\frac{{S}_{m}}{{S}^{0}}+{\text{ln}}\frac{{S}_{m }}{{S}_{m+1}}\right)+{\Psi }_{1}\left({I}_{m+1}-{I}_{m}\right)+ {\Psi }_{2}\left({Q}_{m+1}-{Q}_{m}\right)+{\Psi }_{3}\left({R}_{m+1}-{R}_{m}\right).$$

Using the inequality ${\text{ln}}x\le x-1$, (15) becomes

$$\begin{aligned}\Delta {P}_{m}& \le {S}_{m+1}-{S}_{m}+{S}^{0}\left(-1+\frac{{S}_{m }}{{S}_{m+1}}\right)+\left(-1+\frac{{I}_{m }}{{I}_{m+1}}\right){\Psi }_{1}\left({I}_{m+1}-{I}_{m}\right)\\ &\quad+ \left(-1+\frac{{Q}_{m }}{{Q}_{m+1}}\right){\Psi }_{2}\left({Q}_{m+1}-{Q}_{m}\right)+\left(-1+\frac{{R}_{m}}{{R}_{m+1}}\right){\Psi }_{3}\left({R}_{m+1}-{A}_{m}\right).\end{aligned}$$

16 $$\begin{aligned}&=-\left(1-\frac{{S}^{0}}{{S}_{m+1}}\right)\left({S}_{m+1}-{S}_{m}\right)- \left(1-\frac{{I}_{m }}{{I}_{m+1}}\right){\Psi }_{1}\left({I}_{m+1}-{I}_{m}\right)\\ &\quad-\left(1-\frac{{Q}_{m }}{{Q}_{m+1}}\right){\Psi }_{2}\left({Q}_{m+1}-{Q}_{m}\right)-\left(1-\frac{{R}_{m}}{{R}_{m+1}}\right){\Psi }_{3}\left({R}_{m+1}-{R}_{m}\right).\end{aligned}$$

The value of (16) can be expressed by making use of system (3).

17 $$\begin{aligned}\Delta {P}_{n} & \le -\left(\left(1-\frac{{S}^{0}}{{S}_{m+1}}\right)\left(\vartheta -\beta {S}_{m+1}{I}_{m}-{\mu }_{3}{S}_{m+1}\right)+{\left(1-\frac{{I}_{m }}{{I}_{m+1}}\right)\phi }_{1}\left(\beta {{S}_{m}}_{+1}{I}_{m}-\left({\mu }_{3}+{\gamma }_{3}+{\alpha }_{1}+d\right){I}_{m+1}\right)\right. \\ & \quad + \left. \left(1-\frac{{Q}_{n }}{{Q}_{n+1}}\right){\phi }_{2}\left(d{I}_{m+1}-\left({\mu }_{3}+{\psi }_{1}+{\alpha }_{1}\right){Q}_{m+1}\right)+\left(1-\frac{{R}_{m}}{{R}_{m+1}}\right){\phi }_{3}\left(\varrho {A}_{n}+ \phi {Q}_{n}-\left(\delta +{t}_{1}\right){R}_{m+1}\right)\right).\end{aligned}$$

Let ${\Psi }_{i}$ for $i=\text{1,2},\text{3,4}$ be selected so that

$$\left(\vartheta -\beta {S}_{m+1}{I}_{m}-{\mu }_{3}{S}_{m+1}\right)={\Psi }_{1}\left(\beta {{S}_{m}}_{+1}{I}_{m}-\left({\mu }_{3}+{\gamma }_{3}+{\alpha }_{1}+d\right){I}_{m+1}\right)$$

$${\Psi }_{2}\left(d{I}_{m+1}-\left({\mu }_{3}+{\psi }_{1}+{\alpha }_{1}\right){Q}_{m+1}\right)={\Psi }_{3}\left({\gamma }_{3}{I}_{m+1}+{\psi }_{1}{Q}_{m+1}-{\mu }_{3}{R}_{m+1}\right)$$

By putting the above values, from (17) we get

$$\Delta {P}_{m}\le -\left(\left(1-\frac{{S}^{0}}{{S}_{m+1}}\right)\left(\vartheta -\beta {S}_{m+1}{I}_{m}-{\mu }_{3}{S}_{m+1}\right)+ \left(1-\frac{{I}_{m }}{{I}_{m+1}}\right){\Psi }_{1}\beta {{S}_{m}}_{+1}{I}_{m}-\left({\mu }_{3}+{\gamma }_{3}+{\alpha }_{1}+d\right){I}_{m+1}{+\left(1-\frac{{Q}_{m }}{{Q}_{m+1}}\right)\Psi }_{2}\left(d{I}_{m+1}-\left({\mu }_{3}+{\psi }_{1}+{\alpha }_{1}\right){Q}_{m+1}\right)+\left(1-\frac{{R}_{m}}{{R}_{m+1}}\right){\Psi }_{3}\left({\gamma }_{3}{I}_{m+1}+{\psi }_{1}{Q}_{m+1}-{\mu }_{3}{R}_{m+1}\right)\right).$$

Simple calculations yields

18 $$\Delta {P}_{m}\le -\left(\left(1-\frac{{S}^{0}}{{S}_{m+1}}\right)\left(\vartheta -{\mu }_{3}{S}_{m+1}-\left(1-\frac{{I}_{m}}{{I}_{m+1}}\right){\Psi }_{1}\left({\mu }_{3}+{\gamma }_{3}+{\alpha }_{1}+d\right){I}_{m+1}\right)+\left(1- \frac{{Q}_{m }}{{Q}_{m+1}}\right){\Psi }_{2}\left(d{I}_{m+1}-\left({\mu }_{3}+{\psi }_{1}+{\alpha }_{1}\right){Q}_{m+1}\right)+\left(1-\frac{{R}_{m }}{{R}_{m+1}}\right){\Psi }_{3}\left({\gamma }_{3}{I}_{m+1}+{\psi }_{1}{Q}_{m+1}-{\mu }_{3}{R}_{m+1}\right)\right).$$

As ${S}^{0}=\frac{\vartheta }{{\mu }_{3}}$ which implies ${S}^{0}{\mu }_{3}=\vartheta$. By substituting $\vartheta$ in (18), we obtain

19 $$\Delta {P}_{m}=\frac{-{\mu }_{3}}{{S}_{m+1}}{(\left({S}_{m+1}-{S}^{0}\right)}^{2}-\left(1-\frac{{I}_{m}}{{I}_{m+1}}\right){\Psi }_{1}\left({\mu }_{3}+{\gamma }_{3}+{\alpha }_{1}+d\right){I}_{m+1}+{\Psi }_{2}\frac{\beta \vartheta }{{\mu }_{3}\left({\mu }_{3}+{\psi }_{1}+{\alpha }_{1}\right)}{R}_{0})$$

Hence, if ${R}_{0}\le 1$ then from (19) employs $\Delta {P}_{m}\le 0$ $\forall$ $m\ge 0$. Consequently, ${P}_{m}$ is a ${a}_{n}\ge {a}_{n+1}$. Therefore, $\exists$ constant $P$ such that ${{\text{lim}}}_{n\to \infty }{P}_{m}=P$ which recommends that ${{\text{lim}}}_{n\to \infty }\left({P}_{m+1}-{P}_{n}\right)=0$. From system (3) and ${{\text{lim}}}_{m\to \infty }\Delta {P}_{m}=0$ we have ${{\text{lim}}}_{n\to \infty }{S}_{m}={S}^{0}$. For the case ${R}_{0}<1,$ we have ${{\text{lim}}}_{m\to \infty }{S}_{m+1}={S}^{0}$ and ${{\text{lim}}}_{n\to \infty }{I}_{m}=0,{{\text{lim}}}_{m\to \infty }{Q}_{n}=0.$ Since scheme (3), we succeed ${{\text{lim}}}_{n\to \infty }{I}_{m}=0,{{\text{lim}}}_{n\to \infty }R=0$ and ${{\text{lim}}}_{n\to \infty }{Q}_{m}=0.$ For the situation ${R}_{0}=1,$ we have ${{\text{lim}}}_{n\to \infty }{S}_{m+1}={S}^{0}.$ Thus, from system (3), we obtain ${{\text{lim}}}_{n\to \infty }{R}_{m}=0,{{\text{lim}}}_{m\to \infty }{Q}_{m}=0,{{\text{lim}}}_{m\to \infty }{I}_{m}=0$ and ${{\text{lim}}}_{n\to \infty }{R}_{m}=0$. Hence, ${E}_{0}$ is GAS.

## Conclusions

**Figure 2:**
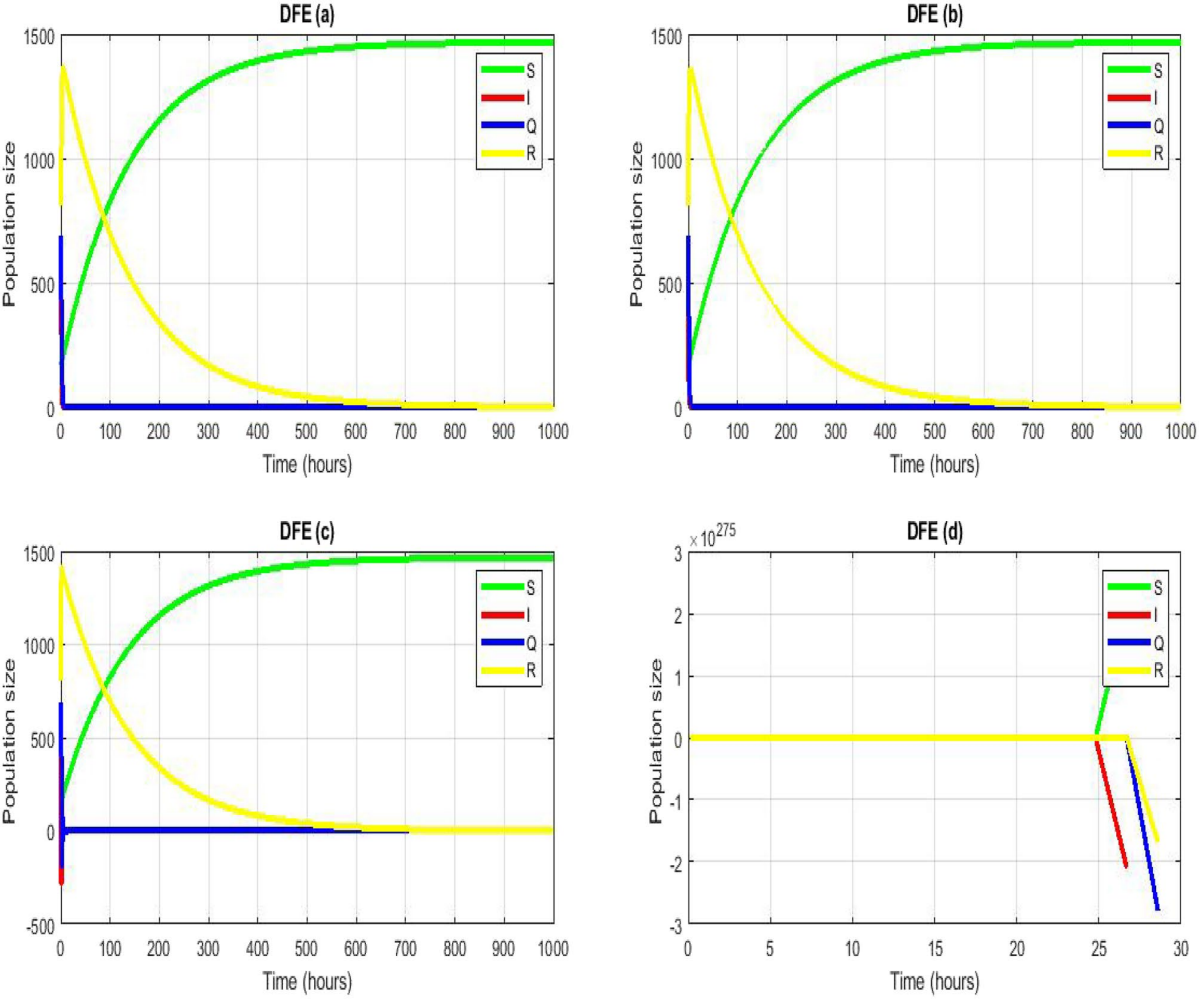
Numerical simulation for model (1) of^30^ by using Euler scheme with $\left(\mathbf{a}\right) h = 0.01,\left(\mathbf{b}\right) h = 0.1,\left(\mathbf{c}\right) h=1,\left(\mathbf{d}\right) h=2$. (**a**–**d**) Stable CFE point $\vartheta =10.48,\beta =0.00714, {\mu }_{3}=0.0004,{\alpha }_{1}=0.42386, d=0.7 , {\psi }_{1}=0.475,{\gamma }_{3}=0.0135$.

**Figure 3:**
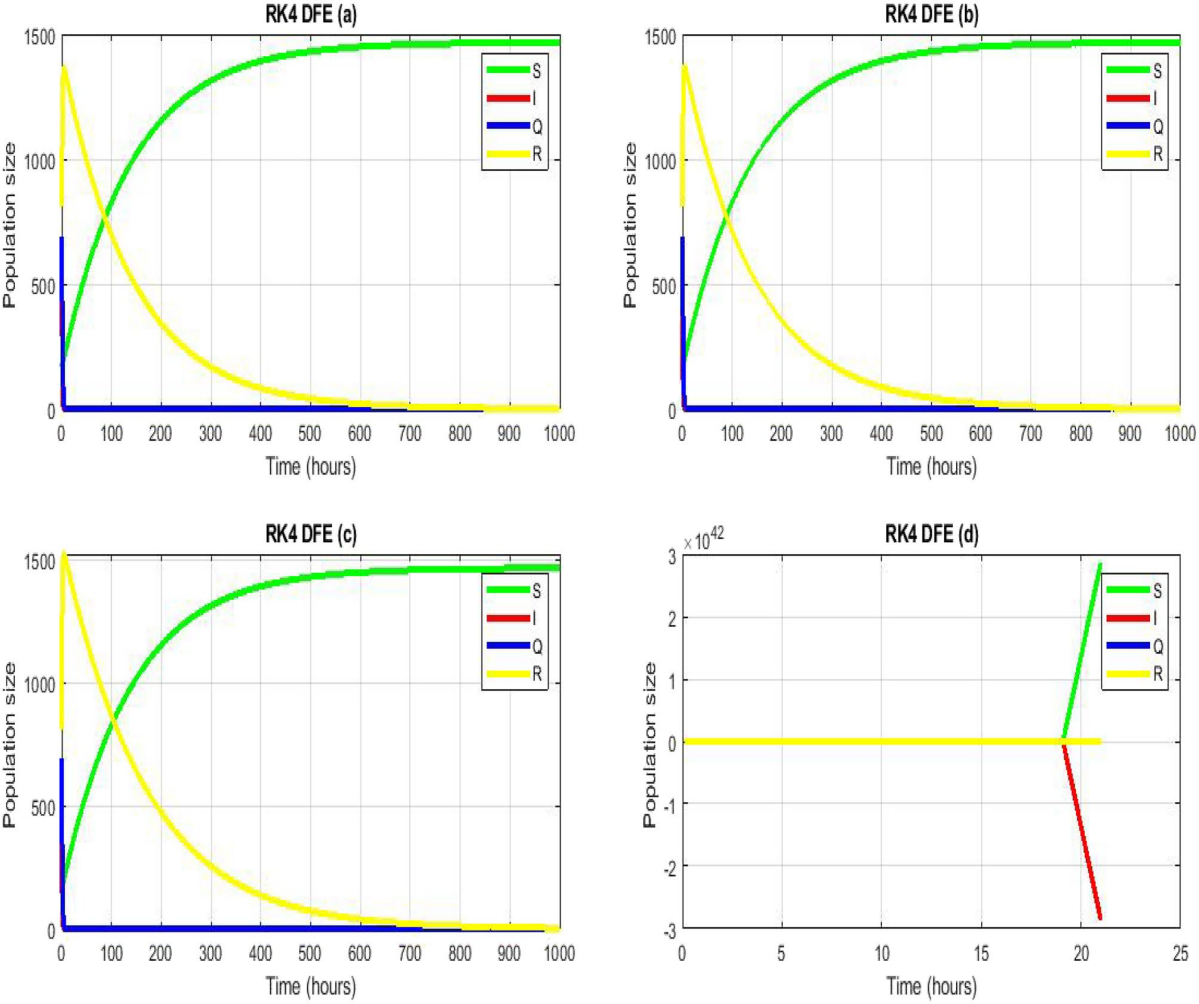
Numerical simulation for model (2) of^30^ by using RK4 scheme with $\left(\mathbf{a}\right) h = 0.01,\left(\mathbf{b}\right) h = 0.1,\left(\mathbf{c}\right) h=1,\left(\mathbf{d}\right) h=2$. (**a**–**c**) Stable CFE point $\vartheta =10.48,\beta =0.00714, {\mu }_{3}=0.0004,{\alpha }_{1}=0.42386, d=0.7 , {\psi }_{1}=0.475,{\gamma }_{3}=0.0135$.

**Figure 4:**
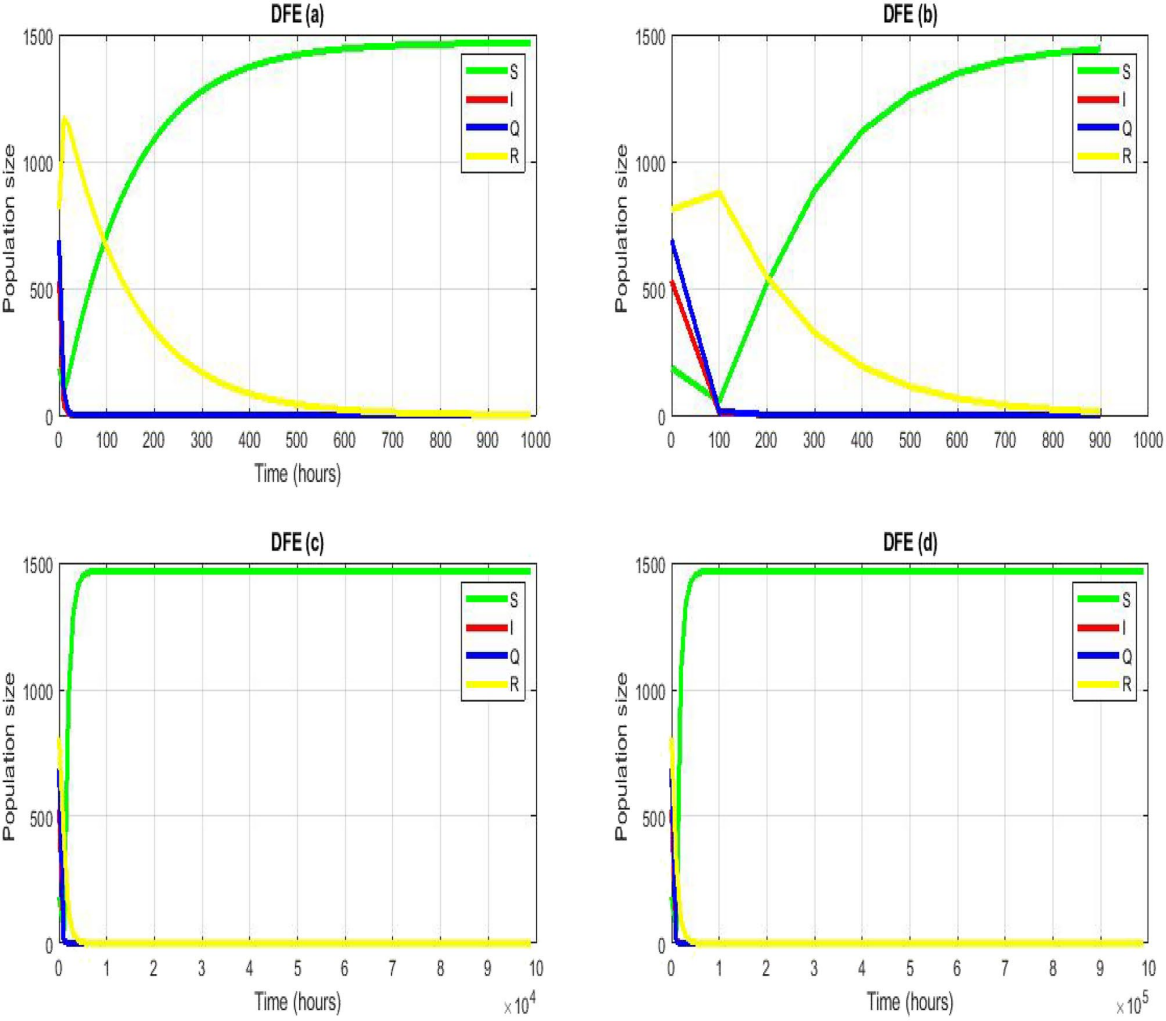
Numerical simulation for model (1) of^40^ by using NSFD scheme with $\left(\mathbf{a}\right) h =1,\left(\mathbf{b}\right) h = 10,\left(\mathbf{c}\right) h=100,\left(\mathbf{d}\right) h=1000$. (**a**–**d**) Stable CFE point $\vartheta =10.48,\beta =0.00714, {\mu }_{3}=0.0004,{\alpha }_{1}=0.42386, d=0.7 , {\psi }_{1}=0.475,{\gamma }_{3}=0.0135$.

In this study, we used a math model to analyze COVID-19, considering both symptomatic and asymptomatic conditions. We set a critical threshold value to explore the stability of key points in the continuous model. For this model, we developed algorithms like Euler, RK-4, and NSFD. Euler and RK-4's reliability depends on step size, with larger steps leading to more unpredictable results. In contrast, NSFD consistently converges regardless of step size. We examined the stability of key points for the NSFD scheme, considering both local and global aspects. By taking monotonic sequences into account, we assessed global stability. The NSFD scheme highlighted similarities between discrete and continuous models, offering advantages for society and medicine. Which we showed in the Figs. 2, 3 and 4. These findings can aid in predicting the course of the COVID-19 pandemic. Numerical simulations were incorporated at each step to support our theoretical framework.

We're studying different ways diseases spread to understand them better. In the future, we plan to improve our model by using sensitivity methods along with the NSFD technique to find the best dynamics behavior for the system.

## Data Availability

The data used to support the finding of this study are included within the article.
